# A novel and sensitive real-time PCR system for universal detection of poxviruses

**DOI:** 10.1038/s41598-021-81376-4

**Published:** 2021-01-19

**Authors:** Léa Luciani, Lucia Inchauste, Olivier Ferraris, Rémi Charrel, Antoine Nougairède, Géraldine Piorkowski, Christophe Peyrefitte, Stéphane Bertagnoli, Xavier de Lamballerie, Stéphane Priet

**Affiliations:** 1 Unité des Virus Émergents (UVE: Aix-Marseille Univ-IRD 190-Inserm 1207), Marseille, France; 2 Centre National de Référence-Laboratoire Expert Orthopoxvirus, Institut de Recherche Biomédicale Des Armées (IRBA), 91220 Brétigny-sur-Orge, France; 3 IHAP, Université de Toulouse, INRAE, ENVT, Toulouse, France

**Keywords:** Pox virus, Infectious-disease diagnostics

## Abstract

Success in smallpox eradication was enabled by the absence of non-human reservoir for smallpox virus. However, other poxviruses with a wider host spectrum can infect humans and represent a potential health threat to humans, highlighted by a progressively increasing number of infections by (re)emerging poxviruses, requiring new improved diagnostic and epidemiological tools. We describe here a real-time PCR assay targeting a highly conserved region of the poxvirus genome, thus allowing a pan-Poxvirus detection (Chordopoxvirinae and Entomopoxvirinae). This system is specific (99.8% for vertebrate samples and 99.7% for arthropods samples), sensitive (100% for vertebrate samples and 86.3% for arthropods samples) and presents low limit of detection (< 1000 DNA copies/reaction). In addition, this system could be also valuable for virus discovery and epidemiological projects.

## Introduction

The successful global eradication of smallpox (caused by the *Variola virus*) in 1980 was an unprecedented victory for humankind and preventive medicine, making the world free of one of its most dangerous diseases. This was achievable due to the adoption of a World Health Organization vaccination strategy, which exploited the absence of a non-human poxvirus reservoir and the cross-reactive immunity induced by the related avirulent strain, viz., *Vaccinia virus*. *Vaccinia virus* and *Variola virus* are both members of the genus *Orthopoxvirus* within the *Chordopoxvirinae* subfamily.

Paradoxically, this triumph created a situation in which the subsequent cessation of vaccination has rendered the global human population vulnerable to (ortho)poxvirus infections. This is illustrated by the gradual increase in numbers of infections by (re)emergent members of the genus *Orthopoxvirus*, including *Monkeypox*^1,2^, *Cowpox*^3^, *Camelpox* and *Buffalopox virus*^4^. Among these viruses, *Monkeypox virus* is the most pathogenic with a case fatality rate of 1–10% and an attack rate of 9% in the 1980s that was recently reassessed to 50% due to a decrease in the prevalence of smallpox vaccine. *Cowpox virus* does not appear to be capable of human-to-human transmission but can infect various hosts, particularly rodents, which, with a sero-prevalence rate up to 70%^5^, seem to have a role in its dissemination. The *Camelpox virus*, whose genome is very similar to that of smallpox, has a mortality rate of 10–30% in camels, but its precise impact on humans has not yet been evaluated. *Buffalopox virus*, which is responsible for localized epidemics in Central-Asia^6^, is yet poorly studied although a sero-prevalence study in human have shown that 17% of unvaccined population have antibodies against *Buffalopox virus*, suggesting a sub-clinical circulation in this region^4^. Other genera can cause human poxvirosis like *Molluscipoxvirus* (prevalence of 5 to 11% in children^7^), the zoonotic *Parapoxvirus*^8^ (common infection in people in contact with infected sheep and cattle), *Yatapoxvirus*^9^ (affecting humans and primates in Africa) or the strain NY_014 recently discovered in an immunocompromised patient which seems to be close to a new genus: *Centapoxvirus*^10^. Poxviruses are ubiquitous, known to infect a broad-spectrum of hosts, and their capacity to switch hosts is only partly understood and is unpredictable^11,12^. These observations imply that (re)emergent poxviruses from several genera could constitute a public health concern in the future. Consequently, there is a need for physicians and veterinarians to be better prepared for the re-emergence of already known poxviruses and for the potential emergence of yet unknown ones.

The diagnosis of known poxvirus infections combines clinical examination and molecular biology. The real-time PCR systems developed to date only enable the detection of poxviruses implicated in human (mainly from the genera *Orthopoxvirus*^13^ and *Parapoxvirus*^14^) and veterinary diseases^15^. Although broader spectrum systems was published in 2010 and 2014, they were conventional PCR system that excludes members from several genera and *Entomopoxvirinae*^16,17^. No qPCR system capable of detecting all poxviruses is therefore currently available. Such a tool would be less burdensome, faster and more accurate than conventional PCR and could meet future needs for diagnosis and discovery of new poxviruses.

Here, we report and validate the performance of a qPCR detection system, targeting a nucleotide sequence conserved in all poxviruses, and capable not only of detecting known pathogenic poxviruses but also any other poxviruses from the *Chordopoxvirinae* and *Entomopoxvirinae* subfamilies.

## Results

**Figure 1:**
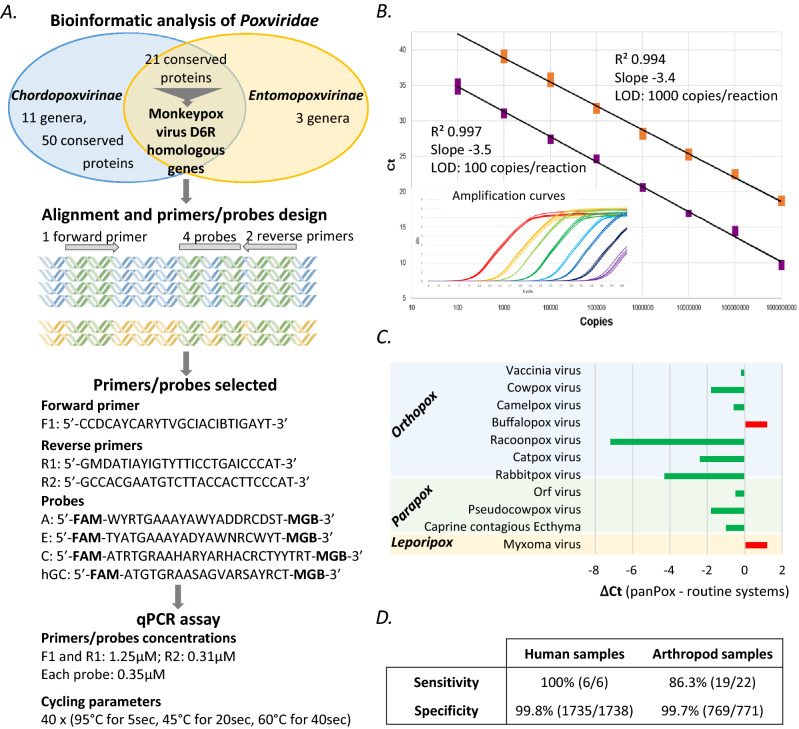
Development of a panPox real-time PCR system. (**A**) Workflow of panPox qPCR assay design. The bibliographic and bioinformatic analysis enables the identification of conserved genes between *Chordopoxvirinae* and *Entomopoxvirinae*. Protein blast of *Chordopoxvirinae* sequences on *Entomopoxvirinae* database identified 21 conserved proteins. Alignment of candidate genes (highest Blast scores) facilitated the design of a qPCR system combining 1 forward and 2 reverse primers and 4 probes and targeting the Monkeypox virus D6R gene and homologs. The DNA icon was obtained from the Pixabay image bank (mcmurryjulie). (**B**) Limits of detection and reproducibility examples. DNA standards corresponding to *the Bovine papular stomatitis virus* (BPSV) (orange squares) and *Monkeypox virus* (purple squares) are shown. The parameters of the standard curve and the limit of detection (LOD) are described next to the standard curve. The insert shows the amplification plot of *Monkeypox virus* standards at 10^9^ (red), 10^8^ (yellow), 10^7^ (light green), 10^6^ (dark green), 10^5^ (light blue), 10^4^ (dark blue), 10^3^ (black) and 10^2^ (purple) copies/reaction repeated in quadruplicate. (**C**) Comparison of the panPox system against Myxoma-specific or panOrthopoxvirus and panParapoxvirus qPCR systems (detailed in Supplemental Methods). The results are expressed as Ct value differences between panPox and routine systems. (**D**) Sensitivity and specificity description on a large panel of human and arthropod samples.

To establish a real-time PCR system able to detect any poxviruses, we first looked for a conserved gene within the entire *Poxviridae* family genome^18^. The Monkeypox virus D6R homologous genes (70 kDa small subunit of early gene transcription factor VETF) were the only ones displaying a conserved region of at least 100 nucleotides suitable for primers/probes design (Fig. 1A and Supplemental Fig. 1 and detailed in the method section). Numerous primers (10 forward and 12 reverse) and 13 probes were designed and evaluated, alone or mixed, on synthetic plasmids containing the sequence of the target conserved region of 22 different virus species among 13 genera of the *Chordo-* and *Entomopoxvirinae* subfamilies. After this optimization process, the combination of one forward and two reverse primers, with four probes gave the best results on all synthetic standards.

The resulting qPCR system, hereafter named panPox, was then assessed for sensitivity on all synthetic plasmids. The detection limit (DNA copies/reaction) was 100 for 12 species and 1000 for the others (Fig. 1B and Supplemental Table 1). High reproducibility during varying test conditions and between operators was demonstrated by the coefficients of variation (CVs) of intra-assay variability lower than 4% (Supplemental Table 1). Similar results for inter-assay variability were obtained (data not shown).

Poxvirus strains and positive clinical specimens (11 different species) were further used to evaluate the efficiency of the panPox assay through Ct value comparison to previously published qPCR systems that are routinely used in French hospitals and veterinarian facilities (Fig. 1C and Supplemental Table 2). As expected, the entire samples positive for a given poxvirus were detected by the panPox system, showing that it has been always as effective as the pan-genera reference systems^13,14^ on species from *Ortho*- and *Parapoxvirus* genera and the extremely sensitive *Myxoma* virus-specific system^19^. Some minor differences (⁓ 1Ct) between panPox and reference systems were observed for *Buffalopox* and *Myxoma* virus but these differences were not considered significant given a qualitative positive/negative assay. In contrast, a significant difference (⁓ 7 Cts) was observed for *Raccoonpox* virus, reflecting the inability of the panOrthopox reference system to detect this strain. Indeed, the alignment of the target site sequences of the reference system (Supplemental Fig. 2), which was designed only for human pathogenic poxviruses, confirmed that *Raccoonpox* virus was not detected by the reference system as effectively as by our system. Human clinical samples (blood and cutaneous) known to be positive for other DNA viruses (n = 26) were also used to demonstrate that there was no cross-reactivity against cytomegalovirus, human adenovirus, Epstein Barr virus, varicella zoster virus, herpes simplex virus and herpesvirus 6. Finally, we also controlled that a non-specific amplification of genomic DNA did not exist when using the panPox system (Supplemental Table 2).

A prospective analysis of a large collection of biological samples, including human samples (n = 1744), from various swabs, biological fluids, and biopsies, and crushed arthropod (ticks and sandflies) samples (n = 793) from the different geographical origins was performed to evaluate the specificity and sensitivity of the panPox assay (Fig. 1D and Supplemental Table 3). Each human sample was tested under routine hospital conditions with panOrtho-^13^ and -Parapoxvirus^14^ systems, revealing 2 cases of Orf virus (also diagnosed clinically). The remaining specimens were presumed negative for poxviruses, except 4 samples with a clinical suspicion of *Molluscum contagiosum*. The positive cases (6/6) of poxvirus were all detected by the panPox system. Among 1738 presumably negative specimens, 98.7% (1716/1738) proved to be negative by the panPox assay and 1.1% (19/1738) displayed doubtful and late positive curves but were non-reproducible in a second PCR and were thus considered negative. Three samples gave late positive and reproducible curves but were negative using NGS sequencing and thus were considered to be false positives. The panPox system therefore had a specificity of 99.8% (1735/1738) on human specimens. Crushed arthropod samples from sandflies (n = 564), cattle ticks (n = 98), wild boar ticks (n = 77) and ticks found attached on patients (n = 54) were then tested using the panPox, and routine panOrtho-^13^ and panParapoxvirus^14^ systems. Among those samples, only cattle ticks displayed some positives (22/98) using a panParapoxvirus system (confirmed by NGS). Most of these parapoxvirus-infected ticks were confirmed by the panPox assay (19/22), but 3 false negatives were obtained and 2/771 (0.26%) false positives were detected by the panPox assay but not by the reference systems and NGS sequencing. The panPox system therefore exhibited a specificity of 99.7% (769/771) and a sensitivity of 86.4% (19/22) on arthropod specimens (Fig. 1D and Supplemental Table 3).

## Discussion

Forty years after the eradication of smallpox, the world of poxviruses remains under-explored, paving the way for a potential threat to human health caused by a (re)emerging poxvirus. The development of rapid and inexpensive molecular biology tools, that can improve our understanding of poxviruses and their epidemiology and also enable the detection of known and yet to be discovered viruses, will benefit both human and veterinary health.

In this study, we have described the first qPCR system, panPoxvirus, targeting a conserved nucleotide sequence amongst all *Poxviridae*. This nucleotide sequence is located within the Monkeypox virus D6R gene, which belongs to the so-called 49 “core” genes of poxviruses. The “core” genes are involved in key functions such as replication, transcription, and virion assembly and are, by definition, present and conserved among all *Poxviridae*, including new variants or emerging viruses^18,20^. In addition, the panPox system used degenerate primers and probes that detect members from the *Entomo*- and *Chordopoxvirinae* subfamilies while being phylogenetically distant. As a result, although recent estimates have suggested a higher point mutation rate for poxviruses than for other dsDNA viruses^21^, the sensitivity and/or specificity of the system should not suffer from potential point mutations. In contrast, pan-genera systems, which are used as routine and are designed only based on human pathogenic poxvirus sequences, can be affected by point mutations. Indeed, these pan-genera systems could thus fail to detect some phylogenetically distant viruses, as it has been observed with *Raccoonpox* virus and the panOrthopox reference system. Altogether, all poxviruses, even those not yet discovered, should be detected by this system. Nevertheless, it is worth noting that the use of degenerate primers and probes tends to increase the limit of detection (LOD). However, as clinical specimens typically contain high loads of poxviral DNA, the LOD does not remain to be a critical factor. Besides, this system demonstrated sensitivity on known poxviruses at least equivalent to previously published generic panOrtho-^13^ and panParapoxvirus^14^ assays in human (sensitivity of 100%) and veterinary samples but not in arthropods (sensitivity of 86%). The panPox also showed no cross-reactivity with other DNA viruses and a specificity higher than 99% for human or arthropod samples. This system could thus be substituted for those used as routine.

To date, the diagnosis of suspected poxvirus infections is carried out through a combination of clinical examination and molecular biology, which only allow, mainly through qPCR, the detection of the genera *Orthopoxvirus*^13^ and *Parapoxvirus*^14^ and certain veterinary diseases^15^. A positive result can be confirmed by a second qPCR to determine the species. However, in case of a negative result, e.g. due to a new emerging poxvirus or in the presence of a non-ortho- or non-parapoxvirus such as members of the genera *Yatapoxvirus*, the use of another often time-consuming technology (such as sequencing or/and electron microscopy) is required. Therefore, even if the panPox system does not enable the identification of the genus or species of the virus, it is a powerful, low-cost, first-line diagnostic tool for the detection of all known poxviruses and, in addition, it could also avoid a false negative result in the event of a poxvirus infection not detected by current routine systems. Furthermore, because of its ability to detect both *Chordopoxvirinae* and *Entomopoxvirinae*, it should also enable the discovery of new species within these subfamilies. A real-time PCR system makes a massive screening program largely affordable and time-saving compared with a metagenomic sequencing approach, thus simplifying the discovery of new species. However, the species of the newly discovered virus will then require a characterization using other biotechnological approach such as sequencing. Finally, the demonstration that this system is usable for veterinary purposes, should ensure early detection of poxviruses in wild, farmed and domestic animals, which may constitute reservoirs. The panPoxvirus diagnostic system could therefore be valuable in preventing zoonotic episodes.

## Materials and methods

### DNA samples

Quantified synthetic plasmids (GenScript) containing the target sequence from the *Monkeypox virus* D6R orthologous genes were used as standards. Confirmed poxviral DNA samples were obtained from the National Reference Centre for Orthopoxvirus (France), the Animal and Plant Health Agency (UK), the veterinary school of Toulouse (France), the Pirbright Institute (UK). Human clinical samples known to be positive for a given poxvirus or other DNA viruses or samples presumed negative for a poxvirus were obtained from the Assistance publique-Hôpitaux de Marseille hospital (APHM, France). All methods were carried out in accordance with relevant guidelines and regulations. All experimental protocols were approved by the Agence Nationale de Sécurité du Médicament et des Produits de Santé (ANSM) under numbers 2012-A01563-40, 2012-A01549-34, 2012-A01589-43, 2012-A01590-43, 2012-A01591-42, 2012-A01593-40, 2012-A01599-34, 2013-A00960-45, 2013-A00961-44, 2013-A00962-43, 2013-A00960-45, 2013-A01536-39, 2015-A00884-45. Written informed consent was obtained from all participants. DNA samples from arthropods (Ticks and sandflies) and the cell lines A549, Vero, BHK-21, PK1, and C6/36, were available at the Unité des virus émergents (France). For viral strains or biological samples, DNAs were extracted by the EZ1 Virus Mini Kit v2.0 (QIAgen).

### Real-time PCR target determination and primers/probes design

To find a conserved target sequence among the entire *Poxviridae* family, the sequences of the 50 proteins known to be conserved among the *Chordopoxvirinae*^18^ were blasted (version 2.8.1 +) on the *Entomopoxvirinae* protein sequences available in GenBank (Fig. 1A). Only 21 proteins displayed similarity amongst the *Poxviridae* family (Supplemental Table 4). The ORF sequence of the first 10 genes with the best E-value was recovered from GenBank for several representative species within each genus of the family *Poxviridae*. Multiple alignments of nucleotide and amino acid sequences were then performed using ClustalW in MEGA 7 software. Only the alignment of the homologs of Monkeypox virus D6R gene revealed a conserved region of at least 100 nucleotides usable for primers (a forward primer F1, two reverse primers R1 and R2) and probes (four probes A, E, C, and hGC) design (Fig. 1A and Figure S1).

The assay was standardized using 3.5μL of samples per assay and the EXPRESS qPCR-SuperMix kit (ThermoFisher) on a QuantStudio-12 K-Flex (Applied Biosystems).

### Real-time PCR assay protocol

The qPCR protocol was standardized using a EXPRESS One-Step SuperScript qRT-PCR SuperMix kit (ThermoFisher), using 1.25 μM of primer F1 and R1 and 0.312 μM of primer R2, 0.375 μM of each hydrolysis probe (A, E, C and hGC), and the following thermal cycling program: 95 °C for 5 min, and then 40 cycles of 95 °C for 5 s, 45 °C for 20 s and 60 °C for 40 s. The reactions were performed in 96-well plates and run on a QuantStudio 12 K Flex real-time PCR system (Applied Biosystems). Samples (3.5μL) were added in a final volume of 10μL. Each assay included negative and positive controls.

### Positive and false positive samples validation

Reference qPCR assays including pan-genera systems for Orthopoxvirus^13^, and Parapoxvirus^14^, and myxomatosis-specific system^19^ were used. For Avipoxviruses detection, standard PCR systems were used^22^. NGS sequencing of the amplicons from the reference PCR assays were also performed.

### Performances of the panPox real-time PCR assay

The performance of the panPox system was evaluated following the recommendations of Vaerman et al.^23^. Amplification efficiency, slope, and R^2^ were determined by the QuantStudio Real-time PCR software (Applied Biosystems). The limits of detection (LOD) for each species were determined by performing 4 replicates with the corresponding plasmid templates diluted tenfold from 10^9^ to 10 copies/reaction. LOD was defined as a > 95% detection rate at a given DNA concentration. The intra- and inter-assay variability were determined using the plasmids appropriately diluted to obtain high and low concentrations of the standards (10^6^ and 10^4^ copies/reaction). Eight of each dilution (high or low) of plasmid were tested per assay. The assay was repeated 3 times on different dates with different operators and separated over several weeks. To evaluate the assay precision, the Ct mean, standard deviations and coefficients of variation (CVs) were calculated. To determine the assay specificity, poxviral and presumed negative human, animal and arthropod biological samples were used. Human samples known to be highly positive for other DNA viruses were also used.

### NGS sequencing

After quantification using Qubit dsDNA HS Assay Kit and Qubit 2.0 fluorometer (ThermoFisher Scientific) of purified PCR products from qPCR assays, libraries were built adding barcodes, for sample identification, and primers using the AB Library Builder System (ThermoFisher Scientific). To ensure equimolar pooling of the barcoded samples, a quantification step was included, using the 2100 Bioanalyzer instrument (Agilent Technologies). An emulsion PCR of the pools and loading on-chip was performed using the automated Ion Chef instrument (ThermoFisher). The S5 Ion torrent (Thermo Fisher Scientific) was used for sequencing following the manufacturer's instructions. A de novo contig was produced using CLC genomics workbench software (Qiagen) and a blast was performed to identify the poxviral species amplified.

## Supplementary information

Supplementary Information.
