# Author Correction: A novel and sensitive real-time PCR system for universal detection of poxviruses

**DOI:** 10.1038/s41598-022-09876-5

**Published:** 2022-04-08

**Authors:** Léa Luciani, Lucia Inchauste, Olivier Ferraris, Rémi Charrel, Antoine Nougairède, Géraldine Piorkowski, Christophe Peyrefitte, Stéphane Bertagnoli, Xavier de Lamballerie, Stéphane Priet

**Affiliations:** 1 Unité Des Virus Émergents (UVE: Aix-Marseille Univ-IRD 190-Inserm 1207), Marseille, France; 2 Centre National de Référence‑Laboratoire Expert Orthopoxvirus, Institut de Recherche Biomédicale Des Armées (IRBA), 91220 Brétigny‑sur‑Orge, France; 3 IHAP, Université de Toulouse, INRAE, ENVT, Toulouse, France

Correction to: *Scientific Reports* 10.1038/s41598-021-81376-4, published online 19 January 2021

The original version of this Article contained an error in Figure 1 where in the R1 primer sequence two nucleotides were missing. The original Figure 1 and accompanying legend appear below.

**Figure 1:**
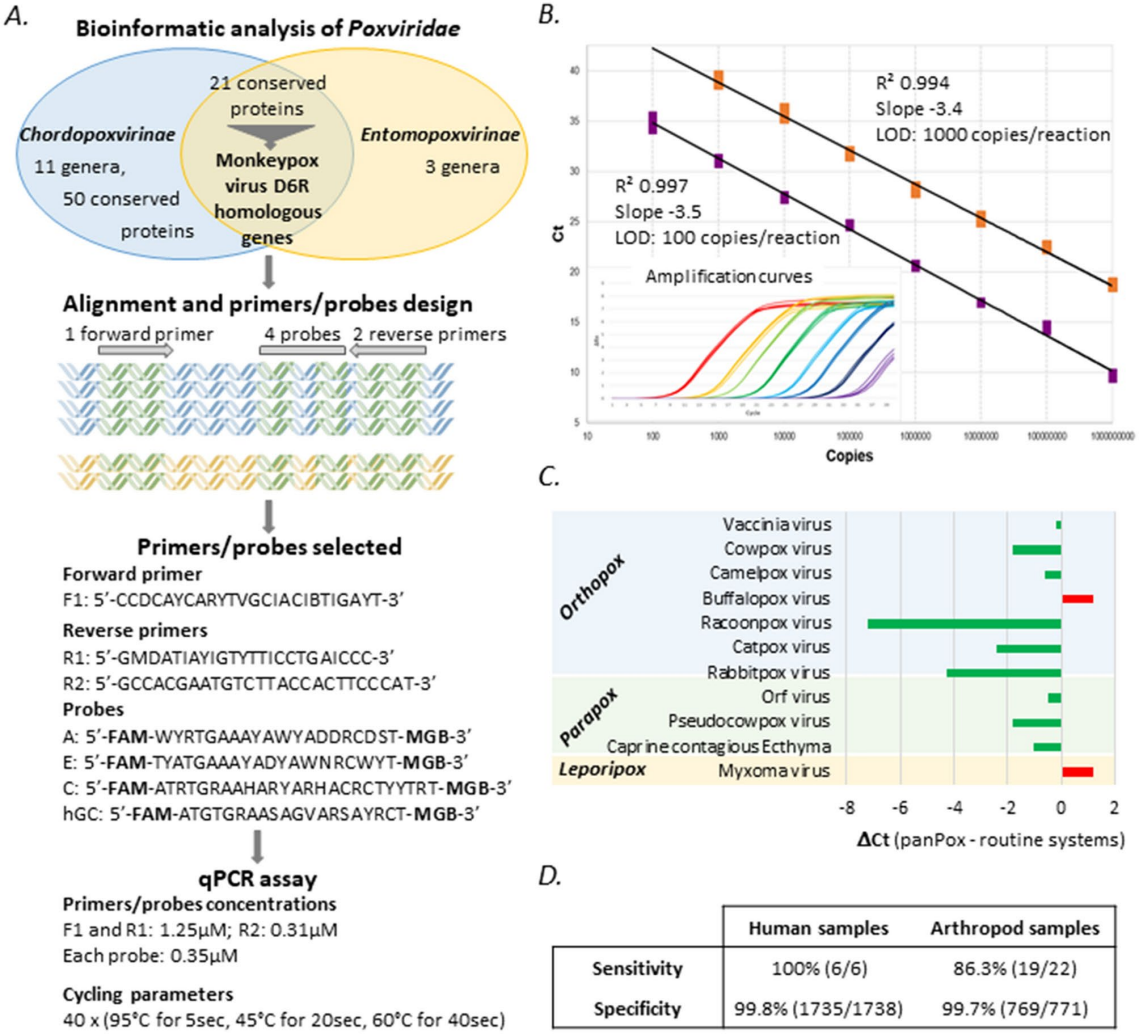
Development of a panPox real-time PCR system. (**A**) Workflow of panPox qPCR assay design. The bibliographic and bioinformatic analysis enables the identification of conserved genes between *Chordopoxvirinae* and *Entomopoxvirinae*. Protein blast of *Chordopoxvirinae* sequences on *Entomopoxvirinae* database identified 21 conserved proteins. Alignment of candidate genes (highest Blast scores) facilitated the design of a qPCR system combining 1 forward and 2 reverse primers and 4 probes and targeting the Monkeypox virus D6R gene and homologs. The DNA icon was obtained from the Pixabay image bank (mcmurryjulie). (**B**) Limits of detection and reproducibility examples. DNA standards corresponding to *the Bovine papular stomatitis virus* (BPSV) (orange squares) and *Monkeypox virus* (purple squares) are shown. The parameters of the standard curve and the limit of detection (LOD) are described next to the standard curve. The insert shows the amplification plot of *Monkeypox virus* standards at 10^9^ (red), 10^8^ (yellow), 10^7^ (light green), 10^6^ (dark green), 10^5^ (light blue), 10^4^ (dark blue), 10^3^ (black) and 10^2^ (purple) copies/reaction repeated in quadruplicate. (**C**) Comparison of the panPox system against Myxoma-specific or panOrthopoxvirus and panParapoxvirus qPCR systems (detailed in Supplemental Methods). The results are expressed as Ct value differences between panPox and routine systems. (**D**) Sensitivity and specificity description on a large panel of human and arthropod samples.

The original Article has been corrected.

