# Supplementary material for: A novel and sensitive real-time PCR system for universal detection of poxviruses
Source: Sci Rep. 2021 Jan 19;11:1798. doi: 10.1038/s41598-021-81376-4 (PMC7815923; doi:10.1038/s41598-021-81376-4)
Supplement: Supplementary file 1 — Supplementary Information. [file 41598_2021_81376_MOESM1_ESM.docx]

**
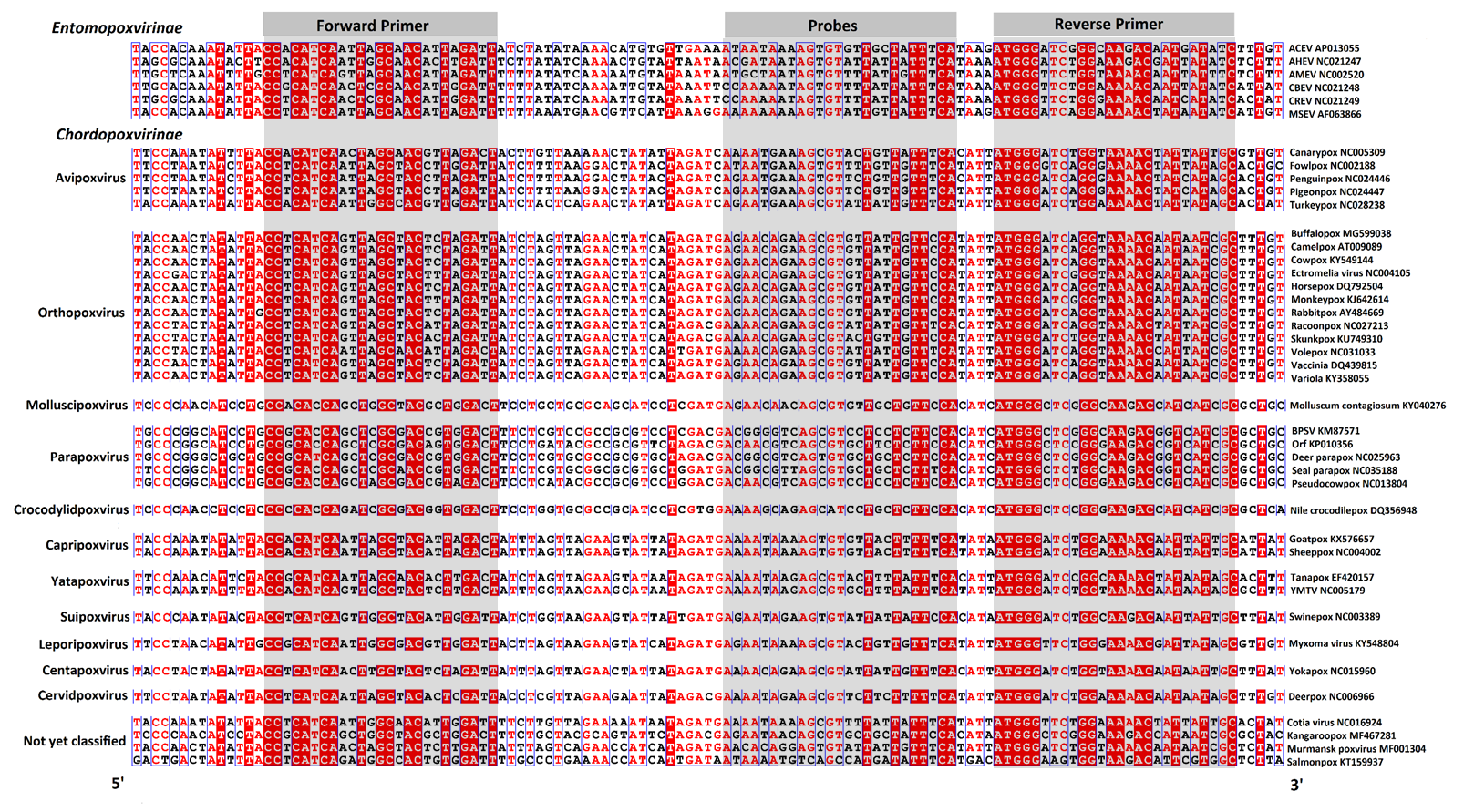
**

**Supplemental Figure 1: Alignment of the panPox target sequence**

Alignment of the nucleotide sequences of the region targeted by the panPox system was performed using ClustalW in MEGA 7 software. The localisation of the primers and probes are indicated as grey rectangles. The exact nucleotide sequence of the primers and probes is depicted in Figure 1A.

ACEV: Anomala cuprea entomopoxvirus, AHEV: Adoxophyes honmai entomopoxvirus, AMEV: Amsacta moorei entomopoxvirus, CBEV: Choristoneura biennis entomopoxvirus, CREV: Choristoneura rosaceana entomopoxvirus, MSEV: Melanoplus sanguinipes entomopoxvirus, BPSV: Bovine papular stomatitis virus, YMTV: Yaba monkey tumor virus


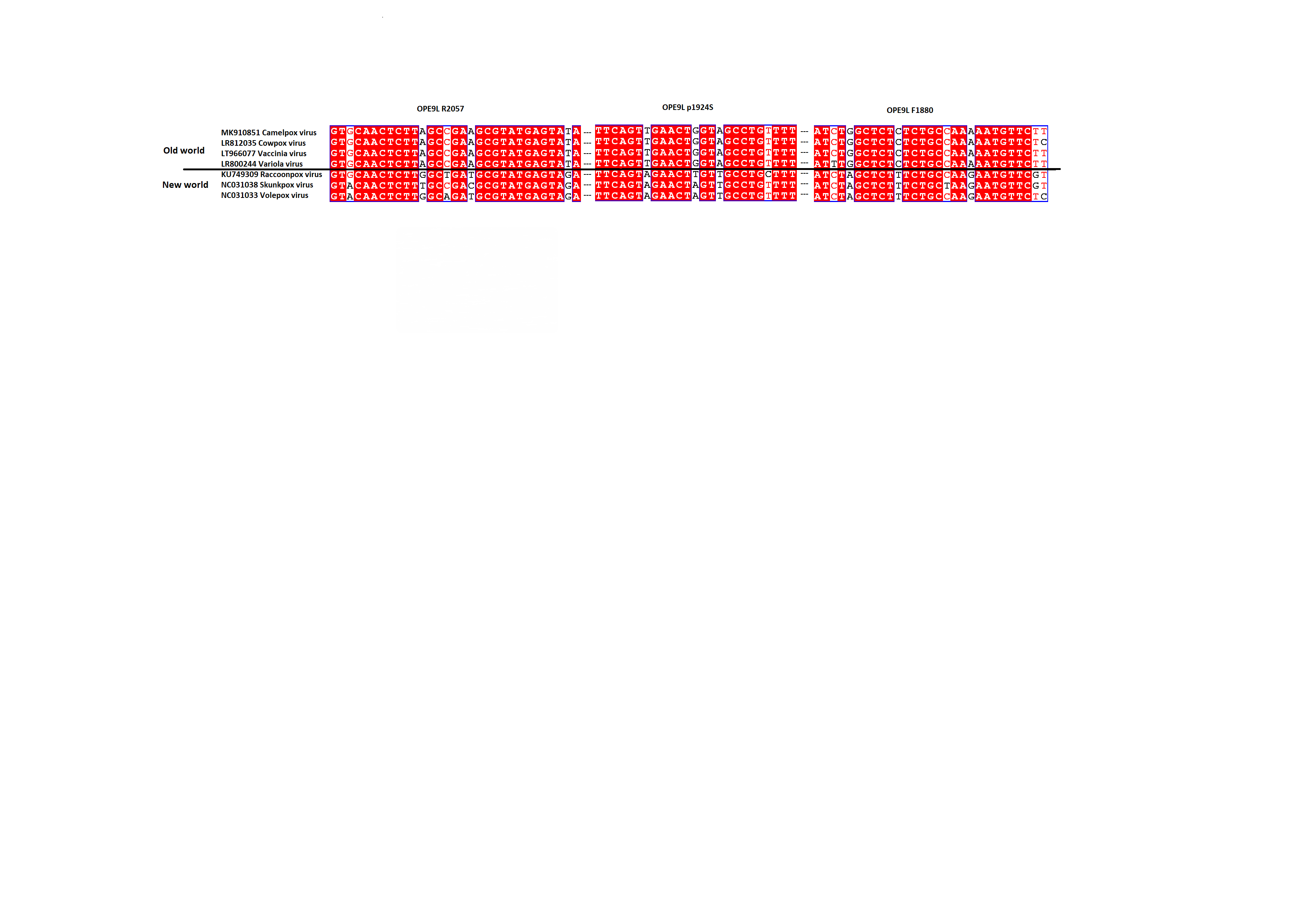


**Supplemental Figure 2: Aligment of the pan-Genus Orthopoxvirus targeting E9L gene**

Detail of the alignment of the E9L gene targeted by the pan-Orthopoxvirus system used for the comparison of the panPox system. This figure highlights the differences between the Old World Orthopoxviruses on which the system was designed and the Old World Orthopoxviruses (including the Raccoonpox virus). This explains the important differences of Ct with our system which targets a more conserved gene which is not impacted by the recent genomic evolution of Orthopoxviruses.

**Supplemental Table 1: Limit of detection determination using DNA standards.**

Description of the target sequence containing plasmids of 22 poxvirus species used to determine the limit of detection (LOD) and the repeatability for high and low copy number. LOD is expressed as copies per qPCR reaction and repeatability was evaluated as the coefficient of variation (Standard deviation (SD) divided by the mean and expressed as a percentage). The parameters of the standard curve obtained (R^2^, Slope and amplification efficiency) are detailed. The coefficients of variation are calculated for high and low concentrations of the standards (10^6^ and 10^4^ copies/reaction).

**Supplemental Table 2: Comparison of panPox and routine systems on poxvirus DNA from strains, human and animal samples.** The origin and type of DNA used is described. Ct values obtained with the panPox and routine systems are depicted. Ct value differences between panPox and the routine test system were calculated.

APHA: Animal an Plant Health Agency (UK)

APHM: Assistance Publique Hôpitaux de Marseille (France)

FNRCO: National Reference Center of Orthopoxvirus (France)

PI: Pirbright Institute (UK)

UVE/EVAg: Unité des virus émergents (France) / EVAg: [www.european-virus-archive.com](http://www.european-virus-archive.com)

VST: Veterinary school of Toulouse (France)

^a^ Suspicion based on clinical examination

Routine systems are from Kulesh *et al* for Orthopoxviruses^b^, Nichte *et al* for Parapoxviruses^c^, Kwit E *et al* for myxomatosis^d^

^e^No qPCR system available

N/A: non-applicable

**Supplemental Table 3: Sensitivity and specificity of the panPox system.**

Biological specimens and their origin are described. The numbers of specimens tested positive with the panPox and routine systems are shown.

^a^Routine systems used are from Kulesh et al for Orthopoxviruses and Nichte et al for Parapoxviruses

**Supplemental Table 4:** **Protein Blast results.**

According to the literature, 49 genes are conserved among the *Chordopoxvirinae*. To find sequences also conserved in *Entomopoxvirinae,* the protein sequences of the 49 candidates from Monkeypox virus were blasted against the *Entomopoxvirinae* protein sequence database. Only 21/49 genes showed conservation; the others produced no results.
